# Image Fiber-Based Miniature Suspended Solid Sensor with High Accuracy and a Large Dynamic Range

**DOI:** 10.1038/s41598-017-17003-y

**Published:** 2017-12-01

**Authors:** Pengfei Qi, Lie Lin, Rui Huang, Sicong Zhao, Haolin Tian, Shuai Li, Qinghe Zhang, Weiwei Liu

**Affiliations:** 1 Institute of Modern Optics, Nankai University, Key Laboratory of Optical Information Science and Technology, Ministry of Education, Tianjin, 300350 China; 2 State Key Laboratory of Hydraulic Engineering Simulation and Safety, Tianjin University, Tianjin, 300072 China

## Abstract

An image fiber-based miniature suspended solid sensor has been demonstrated. The diameter of the sensor is only a few millimeters. A superhydrophobic material is coated on the end of the image fiber to avoid the adsorption of suspended solids and bubbles. Multiple parameters, including mass concentration, morphology and particle sizes of suspended solids, can be visually measured in real time. Dynamic ranges of 0 ~100 kg/m^3^, full range accuracies of ±2‰ and a response time of 0.05 s were experimentally realized for the mass concentration measurements. Determinations of particle sizes of the suspended solids are also presented by means of digital image processing. This new technique will significantly advance ultralow-intrusion measurements in studies on the dynamics of suspended solids.

## Introduction

Suspended solids (SS) are important indicators of water quality and refer to the masses or concentrations of inorganic and organic matter in water bodies^1^. They greatly influence water systems, the effects on which include reducing the penetration of light, altering temperatures, and infilling shipping channels and reservoirs with deposited solids^2,3^. Without timely SS detection and control, SS will further lead to serial undesirable impacts such as reduced aesthetic interest, hindered photosynthesis, blocked channels and decreases in the longevities of dams, reservoirs and other water infrastructures. For example, approximately 0.5 to 1% of global reservoir volumes are lost every year as a result of sedimentation^4,5^. In addition, bottom sediments that provide the habitats and sources of nutrients for benthic organisms inevitably carry toxic materials such as pesticides and metals that readily adsorb to sediment particles^1^. As a consequence, the suspension, transport, and deposition of SS in bays and estuaries possess critical importance for understanding complex water systems^3,6^, which have been considered to be “the challenge of the 21st century”^7,8^.

The monitoring of concentrations and particle size distributions are key issues affecting the modeling and understanding of SS dynamics. Various methods for measuring suspended sediment concentrations have been reported in past years. Because conventional laboratory analysis requires long processing times, it provides poor temporal knowledge of SS transport phenomena^9,10^. Optical turbidity measurements by virtue of Mie scattering theory have been more frequently used recently and potential access to continuous measurements over shorter time steps^11–13^. However, the relationship between turbidity and SS concentration depends on the geometric and optical characteristics of suspended particles and can be influenced by numerous factors such as particle size, air humidity and site-specific issues^14,15^. Ultrasonic backscatter is another widespread method. Converting backscatter intensity to mass concentration is a complicated process and depends on multiple factors that include environmental characteristics such as salinity, temperature, pressure, and instrument response. On the other hand, a number of convenient and less expensive particle sizing techniques involving laser-light scattering have been developed, including laser Doppler velocimetry for particles being carried at constant velocities^16^, the inversion of the extinction spectrum of a static solution^17^ and angular scattering distribution measurements of static suspension particles^18^. However, all of these methods are based on the dependence of optical characteristics on particle size by virtue of Mie scattering. The visualized image method employing a microscope is limited by complicated image sampling and is generally applied to static suspension particles in the lab. More importantly, the sensors in all the above mentioned measurement techniques are so large (~*ϕ*50 × 200 mm for common sensors) that they would strongly impact the dynamic characteristics of a water body. That is, they will introduce undesirable errors in dynamic observations of SS, especially for small volume water bodies used in laboratories to study, for example, turbulence generated by vibrating grids^19,20^ and standard jar tests^21,22^.

Optical fiber sensors allow promising techniques for applications to the remote and continuous monitoring of particle concentrations and sizes with ultralow-intrusion in flowing media. Their use presents many degrees of freedom and various advantages that include miniaturization, water resistance, stability, compactness and low-cost, and they therefore have attracted the attention of many researchers^23–26^. In 2009, Omar and MatJafri reviewed implementations of optical fiber sensors for measurements of water turbidity and concentrations of particles from principle to design^27^. However, in previous research, turbidity or particle concentrations under conditions of low dynamic range have only been measured, which were limited by the angles between light source and detector in fixed configurations. Meanwhile, disturbances in measurements from the adsorption of SS and bubbles on fiber ends cannot be eliminated effectively and handily.

In this paper, an alternative image fiber-based suspended solid sensor is reported. An image fiber, which consists of a bundle of fibers and allows the transmissions of images from an end to the other end^28^, has been used as an SS sensor. A superhydrophobic material is coated on the image fiber end to avoid the adsorption of SS and bubbles. Because the diameter of the image fiber was only 1 mm, it may have been the smallest SS sensor reported on to date. By using this miniature sensor, dynamic ranges of 0~100 kg/m^3^ and full range accuracies of ±2‰ have been experimentally demonstrated for mass concentration measurements of SS. The response time is only 0.05 s, which is much faster than those of commercial sensors (~100 s). In addition, multiple parameters such as particle size, morphology and speed of SS in water bodies can also be obtained in real time by processing captured images. Thus, accurate measurements of concentrations without the influence of particle size can be realized by adopting an optimal calibration relationship based on simultaneously measured particle size.

## Measurement equipment setup

**Figure 1:**
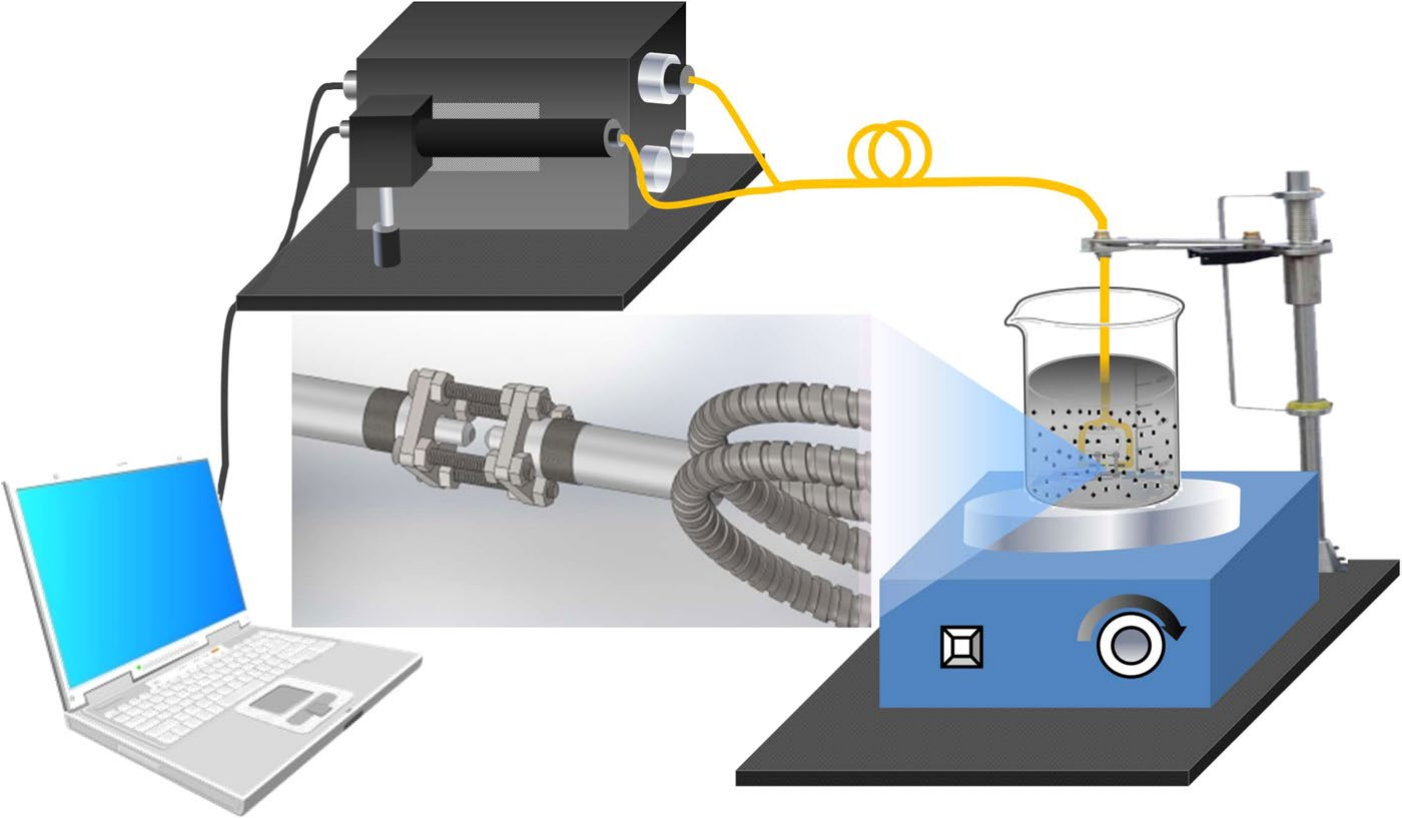
Schematic diagram of the measurement equipment setup.

As shown in Fig. 1, the equipment used to measure suspended solids based on image fibers included four primary parts: a light source, imaging system, and sensor and control systems. In our experiment, an LED with a color temperature of 5000–8300 K was used as a light source. The imaging system consists of a CCD camera and a tunable lens cone equipped with an *f* = 40 mm lens, by which the image at the output end of the image fiber can be magnified. The sensor mainly consists of a light fiber and an image fiber, which are connected with a flange (12.7 mm). There was a gap of several millimeters between the light fiber and image fiber. It is worth noting that the light fiber was made of PMMA organic materials and possessed both good transmittance and flexure resistance such that the core diameter of the light fiber was able to reach 2 mm and fill the need for the uniform illumination of the field of view (FOV), which was impossible for the glass fiber. The image fiber adopted in this equipment had an FOV of 1 mm diameter, and the single fiber diameter and resolution were 13 µm and 44 LP/mm, respectively. A superhydrophobic material was coated on the image fiber end to avoid the adsorption of SS and bubbles^29^. Both kinds of fibers are flexible and can be freely bent to radii below 2 cm. Moreover, the various parameters of the LED and CCD camera were controlled by computer. The fluctuations in the LED light source did not exceed 0.1% when stable, stability being achieved in an hour, and the amount was so small that measurements on a reference radiation were not obligatory. Even so, adding a fiber to monitor the fluctuations of the light source is not difficult for our method if necessary.

During the measurements, the sensor was submerged to a predetermined location in the water body. When the LED and CCD camera were turned on, white light passed through the light fiber and illuminated the measurement location in the water body. The depth of submergence in the water body was approximately 3 cm under a static condition in the experiment, and the upper limit was determined by the image fiber length in the practical application. The image fiber transmitted the sensor images to the input port of the imaging system. The received images were enlarged by the lens and captured by the CCD camera. After imaging processing, multiple parameters, including concentration and SS particle size, were obtained. As limited by the hardware parameters, the equipment operates at speeds of 20 frames per second, which is fast enough to investigate the dynamics of SS. In the experiment, uniform suspensions were maintained in the prepared turbid water bodies by the vigorous stirring of a magnetic stirrer, as shown in Fig. 1.

## Measurements of mass concentration

When the sensor described above is submerged in the water body to be measured, the incident light from the light fiber will be weakened before reaching the image fiber’s end as a result of extinction from absorption and scattering. For a suspension of identical, spherical, non-absorbing particles having a range of sizes, if the concentration of the spheres is not too high, the collective scattering can be approximated as a large number of independent single scatterings such that the intensity of the transmitted light can be written as^30^

1 $$I={I}_{0}{e}^{-\frac{\pi L}{4}\int N(D)Q(D){D}^{2}dD}$$

where *I*_0_ is the intensity of the incident light, *L* is the transmitted distance, *N*(*D*)d*D* is the number of particles per unit volume in the size range of *D* to *D* + d*D*, and *Q*(*D*) is the scattering efficiency (the ratio of the scattering cross-section to the geometric cross-section) for a particle of diameter *D*, which is related to the size and refractive index of the SS.

The relationship between the mass concentration *C*_*M*_ of SS and *N*(*D*) can be described as ${C}_{M}=\frac{\pi }{6}\int N(D)\rho {D}^{3}dD$, where *ρ* is the SS density, which is a constant for specific particles, and *C*_*M*_ is the mass concentration of the suspended solid particles. Substituting it into Eq. (1) and considering the response of the CCD camera, the average gray value of a captured image can be written as

2 $$G=g{I}_{0}{e}^{-L{C}_{M}{D}^{\ast }}+{G}_{b}$$

3 $${D}^{\ast }=\frac{3\int N(D)Q(D){D}^{2}dD}{2\rho \int N(D){D}^{3}dD}$$

where *g* is the conversion coefficient between the gray value *G* and intensity *I* of the CCD camera, and *D* ^*^ is the specific scattering coefficient, which is defined as the ratio of the scattering cross-section to the total particle mass.

**Figure 2:**
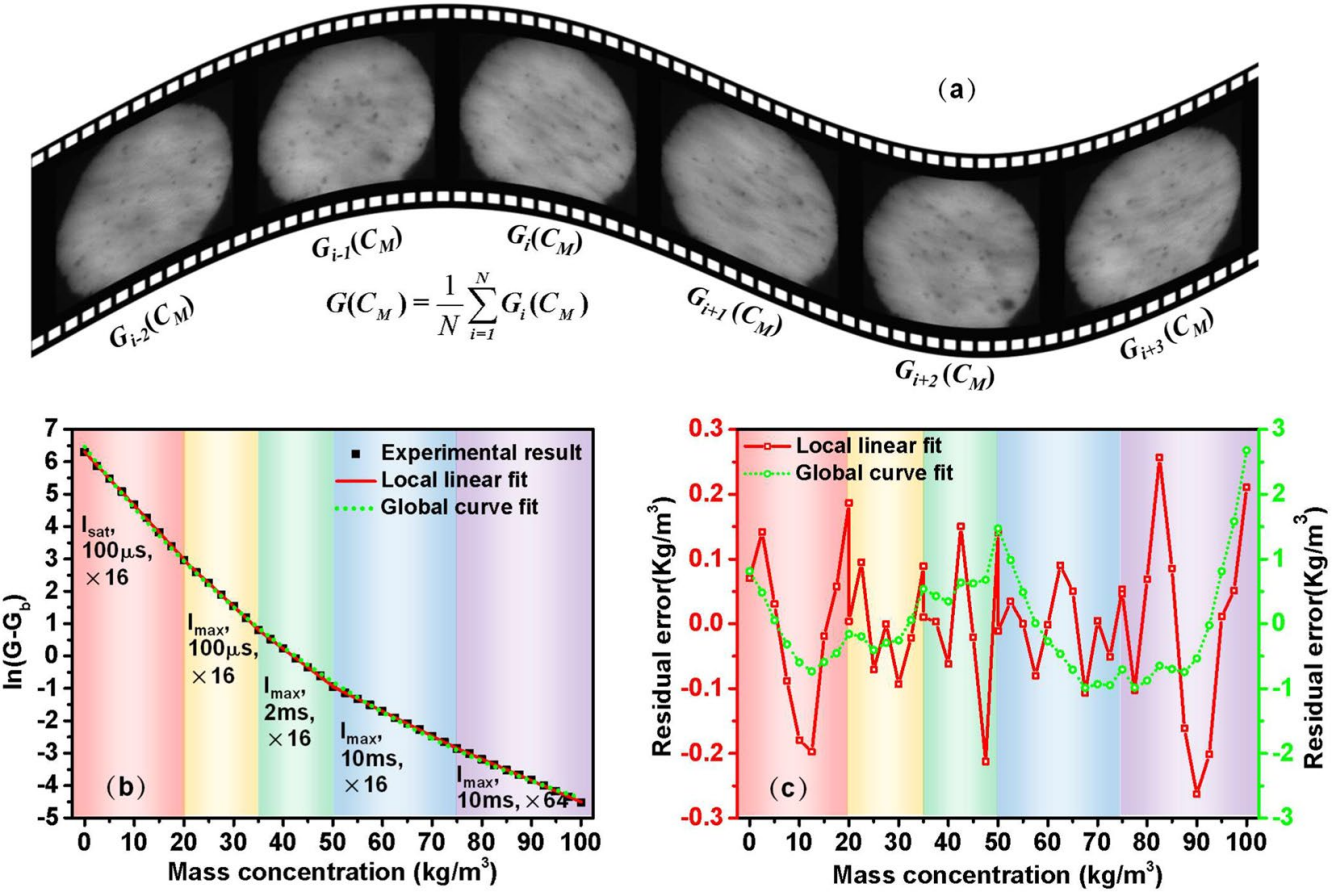
Experimental results and fit lines of the measurements over multiple ranges of SS mass concentrations; (**a**) extraction process of *G*(*C*_*M*_) from the captured images; (**b**) relationship between gray value ln(G-G_b_) and mass concentration over multiple ranges of 0~20 kg/m^3^, 20~35 kg/m^3^, 35~50 kg/m^3^, 50~75 kg/m^3^ and 75~100 kg/m^3^; (**c**) residual errors of the local linear and global curve fits corresponding to (**b**).

The turbid water bodies with different SS mass concentrations were then prepared and maintained in uniform suspension using a magnetic stirrer. At that point, SiC samples were taken using a No. 500 sieve mesh. The CCD gain and exposure time were set to 16 and 100 µs, respectively, and the light intensity was set to *I*_*sat*_ to maintain the CCD near saturation for clear water. The prepared turbid water bodies were then subjected to measurement, and a series of images were captured. To ensure the calibration precision, the average gray *G*_*i*_(*C*_*M*_) of *N* images at each concentration were averaged again, and the final average value $G({C}_{M})=\frac{1}{N}\sum _{i=1}^{N}{G}_{i}({C}_{M})$ was used to calibrate the relationship between the average gray value and mass concentration, as shown in Fig. 2(a). In this experiment, *N* was equal to 500, the relationship between the average gray values and mass concentrations is depicted in Fig. 2(b), and the fitting parameters and determination coefficient *R* ^2^ are noted in Table 1. As shown in Fig. 2(b), the experimental data in the range of 0~20 kg/m^3^ can be perfectly fitted by linear regression, which agrees with Eq. (2), in which the integral term *D* ^*^ is a constant for different mass concentrations with a certain particle size distribution. However, the images became underexposed as the mass concentration of SS increases to 20 kg/m^3^. It is impossible to accurately measure larger mass concentrations in water bodies under such conditions. As we all know, some common advanced optical turbidity meters can also realize measurements over multiple ranges by adding detectors to large sensors to capture the scattering light at different angles; an example is the Turbimax CUS51D, which adopts two detectors to detect scattered light at angles of 90° and 135° for measurements of lower and higher concentrations, respectively. Obviously, such methods for improving measurement range are greatly limited by space and cost.

**Table 1:** Local linear fitting parameters and global curve fitting function corresponding to Fig. 2(b).

Range (kg/m^3^)	Offset	Intercept	Slope	R-Square
0~20	0	6.30974	−0.16591	0.99956
20~35	−2.51114	5.81635	−0.14276	0.99979
35~50	−5.05631	4.85627	−0.11566	0.99937
50~75	−7.11119	2.86963	−0.07627	0.99994
75~100	−8.62731	2.09986	−0.06602	0.99956
0~100	ln(*G* − *G*_b_) = *14.357exp*(−0.0141*C*_*M*_)−7.895			0.99943

Owing to the adjustable LED light source and CCD camera, ultra large measurement ranges of mass concentrations can be achieved easily using our method and without increased cost. As shown in Fig. 2(b), the incident light intensity and CCD parameters such as gain and exposure time are labeled in the corresponding measurement ranges using different background colors. First, the light intensity of the LED was adjusted to a larger value *I*_*max*_ so that the gray value corresponding to 20 kg/m^3^ was enhanced nearly eightfold. As shown in Fig. 2(b), the experimental data can also be linearly fitted, and the measurement range can be improved to 35 kg/m^3^. It is worth noting that the coefficient *gI*_0_ only has an impact on the intercept of the fit line, based on Eq. (2). To reveal the global regularity of the ultra-large measurement range, the gray value ln(*G*-*G*_*b*_) at 20 kg/m^3^ can be adjusted to an equal value by entirely offsetting the gray values of 20~35 kg/m^3^; the offset values are shown in Table 1. When light intensity is at a maximum, camera parameters such as exposure time (*T*_*exp*_) and gain are alternative means to continually improve measurement range. As shown in Fig. 2(b), the mass concentration measurement range can be greatly improved to 100 kg/m^3^, and the linear fitting remained highly effective with the experimental data. The residual errors, which are defined as the deviations of predicted and true values, are depicted as a red solid line in Fig. 2(c).

It can be observed in Fig. 2(b) and Table 1 that the slopes of the fit lines in the different measurement ranges obviously differed, unlike those of the single scatter model described by Eq. (1). This can be attributed to increases in multiple scattering with increasing mass concentrations of SS. The experimental data in all ranges can be globally fitted using the single exponential function recorded in the last line of Table 1. The fitting curve and residual error are depicted using green solid lines in Fig. 2(b) and (c), respectively. Although the residual error of the global fitting was an order of magnitude larger than the local linear fitting, the *R* ^2^ of 0.99943 indicates that the complicated multiple scattering was well described by the global fitted empirical equation in Table 1.

## Influence of particle size distribution

It is well known that the scattering properties of SS are defined through Mie theory with parameters of the complex refractive index *m* = *n + ik* of a material relative to the surrounding medium and the dimensionless size parameter *πD*/*λ*, where *D* is the particle diameter, and *λ* is the incident light wavelength^31^. For the SiC particles in the water and given light source, the influence of particle size on the scattering properties of SiC can be described by Eq. (3). The scattering efficiency *Q* initially increases with diameter *D* and then undergoes a series of oscillations before settling down to a constant for particles large compared to the wavelength of the light^30,32^. Eq. (3) can be rewritten as

4 $${D}^{\ast }=\frac{3Q}{2\rho {D}_{eff}},\,{\rm{and}}$$

5 $${D}_{eff}=\frac{\int N(D){D}^{3}dD}{\int N(D){D}^{2}dD}$$

where *D*_*eff*_ is referred to as the effective particle size by area^33^.

**Figure 3:**
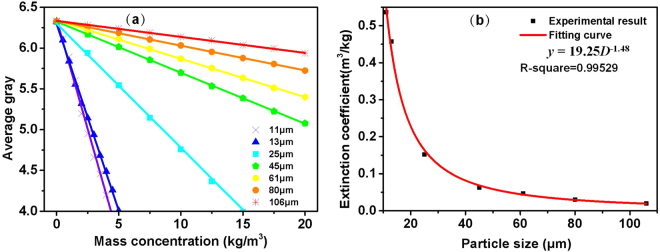
Influence of particle size on measurement of SS, (**a**) experimental results (scattered points) and fit lines (solid lines) of SS for different sieving center diameters, (**b**) extinction coefficients of unit concentrations of SS with different sieving center diameters (scattered points) and fitting curve (solid curve).

To investigate the influence of particle size on the extinction coefficient of unit concentration, the seven SiC samples obtained using different sieving center diameters were measured in the experiment. In addition, the relations between the average gray values and mass concentrations of these SiC samples are depicted in Fig. 3(a), which can be well fitted by the solid lines. The slope of the fitting line corresponding to the extinction coefficient per unit mass concentration can be well fitted by a power function with a power value of −1.48, and the *R* ^2^ is 0.99529, as shown in Fig. 3(b). The deviation of the power values between the fitting function and Eq. (4) can be attributed to the different definitions of the effective particle size by area *D*_*eff*_ and sieving center diameter *D*_*c*_. As depicted in Eq. (5), *D*_*eff*_ is determined by the particle size distribution rather than the center diameter or average diameter. In brief, the particle size distribution of SS greatly influenced the calibration curve.

## Measurements of particle size distribution

The particle size distribution of suspended solids is of primary importance in developing an improved understanding of sediment-water interactions^33^ and is critical to the correct estimation of true sediment concentrations in the field and for model evaluation^34^. The image technique is a visual and convincing method among the various measuring methods for determining SS particle size distributions. However, the difficulties of real-time image acquisition using a minimized probe have vastly limited its applications. With the rapid increase in computer processor power in the past few years, the image technique has been widely used in kinds of detection processes. By combining image processing techniques with our SS sensor, the image technique can be developed into a convenient, rapid and low-cost method for measuring SS particle size distributions.

**Figure 4:**
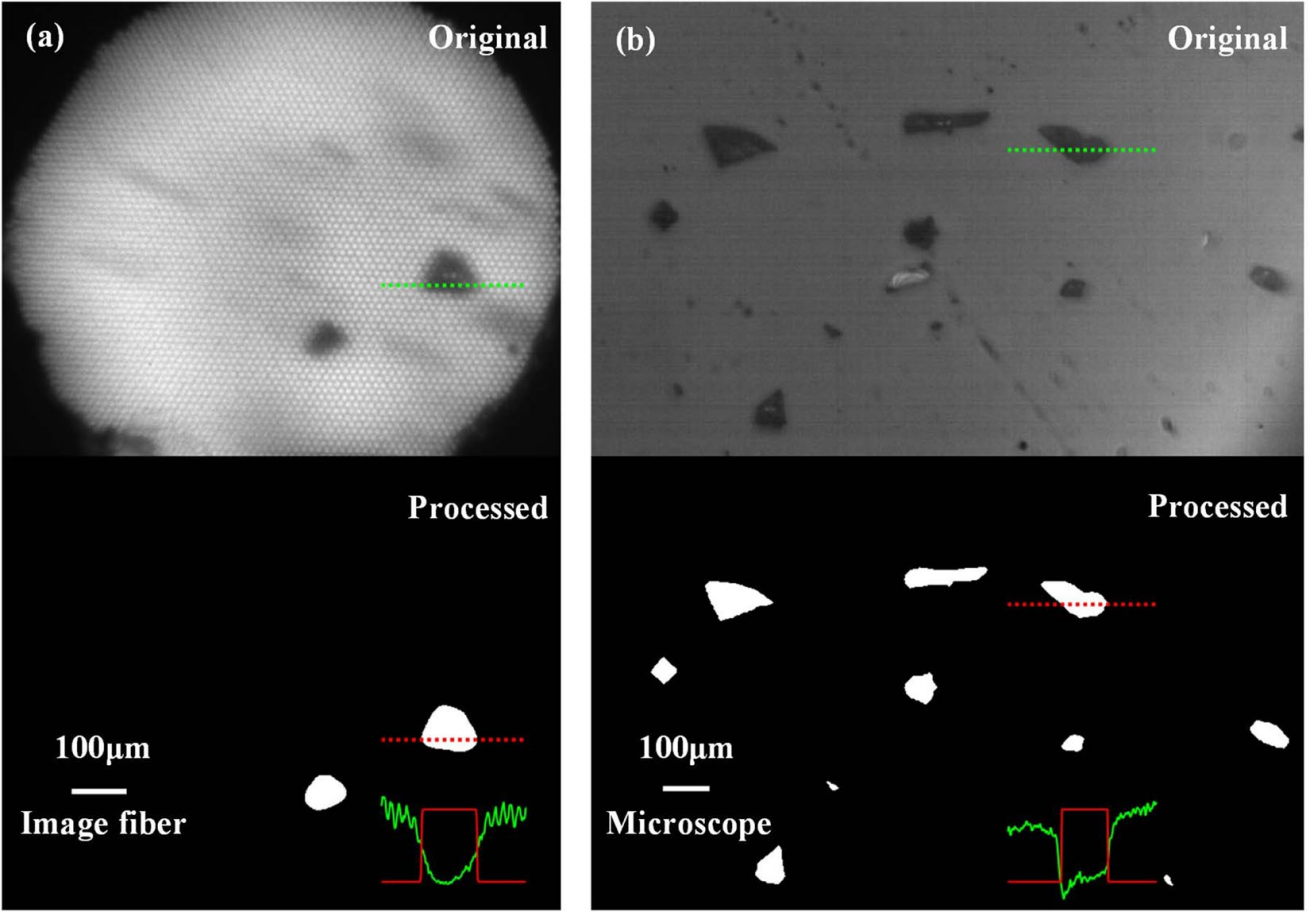
The original and processed images of (**a**) the measuring equipment based on image fiber and (**b**) the microscope, the SiC with sieving mesh of 240 is taken as an example. The green and red solid curves show the profiles of the gray values along the corresponding dotted lines.

**Figure 5:**
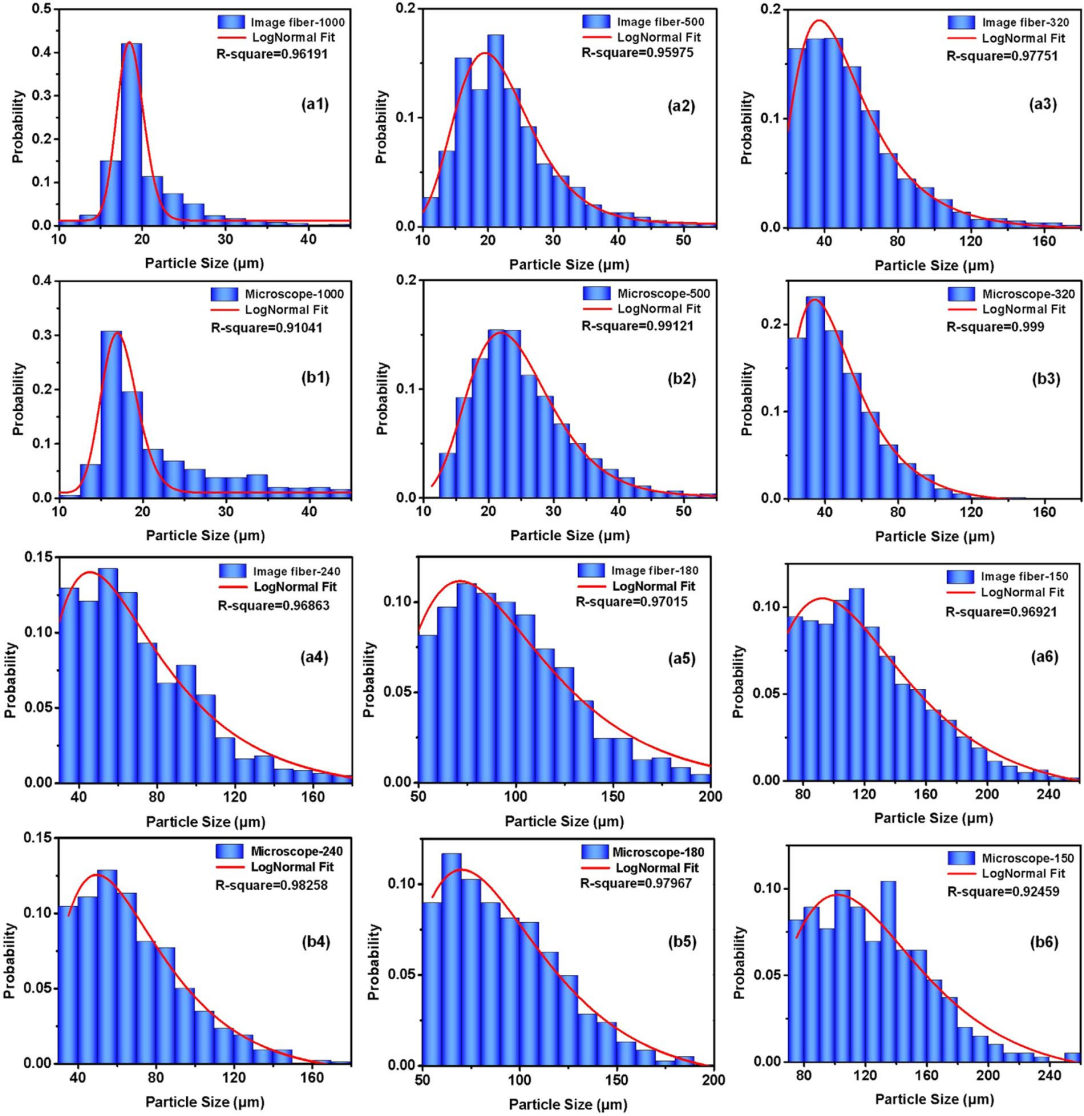
Measurements (blue histograms) and lognormal fitting results (red solid lines) of particle size distributions using the equipment based on the image fiber and microscope.

The images of different SiC particle size distributions can be captured by the operation mentioned above. To reduce the difficulty of image processing, the mass concentrations of samples should not be so high that particle agglomeration occurs. The empirical criterion is that the average gray value corresponding to the mass concentration should be controlled to exceed half of the average gray value of clear water in Fig. 3(a). In some complicated situations, flocculation is also an important factor that cannot be ignored in particle sizing and is mainly affected by, for example, the turbulence intensity^35,36^, pH and salinity^37,38^ of the water. Using image processing procedures such as background correction, contrast enhancement, threshold segmentation, and morphological processing, including dilating, eroding and filling, the diameter of the equivalent circle and morphology of a particle can then be extracted, as shown in Fig. 4. Similarly, the SiC particle size distributions for different sieving meshes can be acquired by the statistics of sufficient particles, as depicted in the histograms shown in Fig. 5(a1)–(a6).

It is worth noting that misinterpretations of solid matter size and morphology away from the image fiber can be avoided for this equipment owing to the short depth of field (DOV) of the image fiber. Specifically, particles away from the image fiber cannot form a clear image for transmission to the distal end and can be easily eliminated from the statistics of particle size using the image processing mentioned above, as shown in Fig. 4(a). Furthermore, the particles far from the image fiber also served as a uniform diffuser for the homogenizing illuminant, which is favorable for imaging. Moreover, the smearing of moving particles in images should be considered in the image-based particle sizing process. The CCD exposure time in the particle size measurements was set to 10 μs, which can avoid smearing in most application cases. For example, particle settling speeds are usually on the order of mm/s^34,39–41^ in observations of sediment transport dynamics, and the particle displacement was ~0.01 μm during the exposure time such that smearing could be neglected when compared with that for particle sizes of several tens of micrometers. The smearing of the moving particles away from image fiber end in the example image can also be easily eliminated using the threshold segmentation image processing procedures, as shown in Fig. 4(a).

It is well known that measured particle size distributions obtained by different methods cannot be cross-referenced. To demonstrate the effectiveness of the equipment based on an image fiber, the microscope was used to capture static particle images for use as references. The corresponding particle size distributions can also be acquired using the same image processing, as shown in the histograms of Fig. 5(b1)–(b6). The particle size distributions of different sieving meshes measured using our instrument and microscope were fitted using the lognormal distribution, which is the common distribution curve used in sizing assays. The lognormal distribution can be described by

6 $$y={y}_{0}+\frac{A}{\sqrt{2\pi }wD}{e}^{-\frac{{(\mathrm{ln}(D/Dc))}^{2}}{2{w}^{2}}}$$

where *D*_*c*_ and *w* are the location parameter and scale parameter, respectively. In Fig. 5, the fitted curves are depicted by red solid lines. The fitted values of each parameter in Eq. (6) for the SiC for different sieving meshes are shown in Table 2. Furthermore, in order to quantitatively describe the conformance of the measuring results based on the two methods, the fitted curve conformities of each mesh are depicted using the Spearman correlation coefficient, which becomes 1 when the two variables are perfectly monotonically related. It can be seen that the measurement results of the two methods were in good accordance, as shown in Fig. 5 and Table 2. The measurement accuracy for particles sizing can also be succinctly expressed by the difference between the equipment and microscopic *D*_*c*_ results, as shown in Table 2. The error can be attributed to the increased blurring of particle edges compared with the static microscope images and the overlapping particles near the end of the image fiber with increasing concentrations. To describe the blurring quantitatively, the profiles of the gray values along the corresponding dotted lines are drawn as green and red solid curves in Fig. 4. It can be determined that the blurring level was approximately the diameter of a single fiber, which is defined as the distance between the positions of the half depths of the valleys and the nearest minimum of the quasi-periodic oscillation curves. In the particle sizing process, the segmenting threshold is adopted as the gray value corresponding to the half depth of the valley to reduce the error from blurring.

**Table 2:** Lognormal fitting parameters corresponding to Fig. 5.

Parameters Mesh	Image fiber				Microscope				Error in *D*_*c*_	Conformity (Spearman Corr.)
	*y*_0_	*D*_c_	*w*	*A*	*y*_0_	*D*_c_	*w*	*A*		
1000	0.012	18.594	0.086	1.650	0.010	17.195	0.124	1.565	8.14%	0.97113
500	0.003	21.224	0.289	2.308	0.001	23.685	0.284	2.439	10.39%	0.95646
320	−0.001	47.959	0.508	10.282	−0.005	43.911	0.496	11.252	9.22%	0.9982
240	−0.006	64.523	0.591	11.738	−0.008	64.474	0.515	9.766	0.08%	0.99528
180	−0.002	89.418	0.477	10.805	−0.010	86.715	0.462	10.607	3.12%	0.99956
150	−0.008	113.498	0.451	13.138	−0.009	121.078	0.417	12.249	6.26%	0.98324

It is also worth noting that the image method based on an image fiber is limited to 2 dimensions, similar to that of most particle size analysis methods such as sieving and micrographs. Due to the random motions of suspended solids, the particle sections measured in the experiment are also random. Although information in the third dimension cannot be acquired, precise size distributions still can be obtained if the number of counted particles is sufficiently large due to the capture of large numbers of images. Furthermore, although opaque SiC has been used as an example for particle size analysis, for this particle sizing technique, the ultralow gray values at the particle locations are due to both the opacity and low fiber coupling efficiency. In consequence, as the transparencies of measured particle increase, the image contrast will decrease but still can be resolved, unless the solids are both transparent and have refractive indexes close to that of the surrounding liquid.

In addition to the fundamental importance of understanding the role of particle sizes in a variety of dynamic processes, particle sizes are also critical to the accurate measurement of concentrations in complex and often highly variable water systems. As previously mentioned, particle size distribution greatly influences the relationship between turbidity and concentration of SS. This implies that there inevitably exists a complicated field or laboratory calibration process before or after mensuration in water area with different SS particle sizes^42^. However, considerable spatial and temporal variabilities in particle size characteristics may occur within relatively small areas and over short times^33^, so that concentrations measured using common optical methods without measurements of particle sizes considerably and negatively impact studies of the dynamic processes of SS. For the presented measuring method that is based on an image fiber, the relationships between turbidity and SS concentration for different particle size distributions can be calibrated, and the optimal calibration relationships can then be adopted in advance according to the measured particle sizes, and therefore, accurate measurements of concentrations without the influence of particle size can be realized.

## Summary and future work

In summary, an image fiber based miniature suspended solid sensor has been demonstrated. A feature of the sensor is its millimetric size. Ultralow-intrusion and real-time measurements of multi-parameters such as mass concentration, particle size, and morphology have been realized. Dynamic ranges of 0~100 kg/m^3^, full range accuracies of ±2‰ and response times of 0.05 s for mass concentration measurements can be easily achieved without increases in cost. In addition, the influence of particle size distribution on the extinction coefficient of unit concentration has been investigated. Measurements of particle size and morphology have also been presented by means of digital image processing. When used in combination with measurements of particle size, accurate measurements of concentrations without the influence of particle size can be realized.

Although a convenient, credible and low-cost method for observations of SS dynamics in complex water systems has been systematically proposed, additional detailed research arising from this work remains worth pursuing. First, in some special applications, the size distributions of particles are more complex, and broad ranges and multiple peaks may appear. Generally, the range of particle size measurements is limited by the diameter of the single fiber (13 µm) and FOV of the image fiber (*ϕ* = 1 mm) so that range-extending is a main optimization target in the future, which can be realized by adopting an image fiber with high resolution or the amplification imaging method using a micro lens and fiber optic taper. For particles with sizes in the measurement range, multiple peaks can be easily measured using such an image method. Second, it is known that blurring can be seen as the result of the convolution of the point spread function and the clear image such that the optimized deconvolution arithmetic for deblurring in digital image processing is significant for improving particle sizing precision. Finally, the image-based method for particle speed detection is also planned research, based on the phenomenon that motion blur is determined by the relative motion between the camera and moving particles during exposure times.
